# In vitro evaluation of probiotic potential and enzymatic profiling of *Pichia kudriavzevii* Y33 isolated from traditional home-made mango pickle

**DOI:** 10.1186/s43141-022-00416-2

**Published:** 2022-09-09

**Authors:** Prem Lata, Reena Kumari, Kiran Bala Sharma, Shailja Rangra, Savitri

**Affiliations:** Department of Biotechnology, Himachal Pradesh University, Summer Hill, Shimla, 171005 India

**Keywords:** Fermented foods, *Pichia kudriavzevii*, Phytase, Probiotics, Traditional pickle

## Abstract

**Background:**

Fermented foods are the results of metabolic activities of various microorganisms. People have traditionally known how to culture desirable microorganisms, primarily lactic acid bacteria, yeasts, and filamentous molds, for the manufacture of edible foods. Yeast isolated from home-made mango pickle from Hamirpur, Himachal Pradesh, was assessed for probiotic properties and their enzymatic profiling.

**Results:**

Four yeast isolates were isolated out of which *P. kudriavzevii* Y33 was selected on the basis of high acid tolerance as well as broadest antimicrobial activity. The selected isolate was observed to have high acid tolerance at pH 2 and show strong antimicrobial activity against all the pathogens examined. *P. kudriavzevii* Y33 can also withstand high bile concentration and showed high viability index, i.e., 95% at concentration of 2% of bile. The isolate was able to demonstrate high cholesterol assimilation in medium containing ox bile and taurocholate, at 88.58 and 86.83%, respectively. The autoaggregation ability of isolate increases with increasing the time of incubation and showed 87% of autoaggregation after 24 h of incubation. *P. kudriavzevii* Y33 exhibited resistance towards different antibiotics, found to be positive for exopolysaccharide production and showed no hemolytic activity. The isolate was observed to produce several enzymes such as β-galactosidase, protease, amylase, phytase, and lipase.

**Conclusions:**

The results of the current study revealed that *P. kudriavzevii* Y33 has various beneficial qualities that suggest it could be used as probiotics. Enzymes produced by yeast isolate help in improving flavor and mineral availability in the fermented products.

## Background

Food fermentation has long been regarded as a cost-effective and practical method of preserving and improving the shelf-life, organoleptic, and functional qualities of perishable food bio-resources [39]. Pickling is an ancient culinary technique of preserving food in brine and/or vinegar that dates back to 2400 BCE. It has long been a part of all societies and cultures around the world and was utilized for food preservation by many ancient civilizations, including the Egyptians, Chinese, and Indians [6].

In India, there is a great deal of diversity in the culinary preferences of individuals living in different states, and many of them, particularly the mountainous ones, have their own traditional fermented products. Himachal Pradesh is known for its various traditional and distinctive pickles, prepared from indigenous fruits grown and available in the state, such as pear, *lasura*, peach, mango, *dheu*, and citrus fruits. Green fruits can be used to make pickle or chutney or as an acidifying condiment (*amchur*), whereas ripe fruits are used to make preserves, jams, sauces, and other foods. Bacteria, yeasts, and fungi are the microorganisms found in traditionally fermented pickles. Some of the yeast species that have been discovered in traditional pickles are *Saccharomyces*, *Geotrichum*, *Candida*, *Debaryomyces*, *Brettanomyces*, *Pichia*, *Kluyveromyces*, *Yarrowia*, *Rhodotorula*, *Cryptococcus*, *Torulopsis*, *Saccharomycopsis*, *Zygosaccharomyces*, and *Schizosaccharomyces* [47].

Probiotics are live microorganisms that provide health benefits to the host when given in proper dosages [18]. A successful probiotic strain is expected to have several desirable properties in order to be able to exert its beneficial effects. The capability to attach to the gastrointestinal mucosa, resistance to gastrointestinal conditions (i.e., gastric acid and bile), and anti-pathogenic capabilities and safety are frequently used as criteria for the selection of probiotics [36]. The selected strain must also be species recognized, strain typed, and evaluated for toxicity, pathogenicity, or any hazardous metabolic activities [52].

The potentiality of yeasts as probiotics in traditional fermented foods is currently less explored as compared to bacterial probiotics, despite the fact that few researchers have investigated the potentialities of probiotic yeasts in food fermentation [22, 27, 43]. The ability to provide consumers with a health benefit is the final criterion for selecting a successful probiotic strain. The tolerance of yeast to antibiotics, the ability to maintain viability in the human gut during antibiotics treatment, and the ability to minimize pathogen adherence to mucosal surfaces are all advantages of utilizing yeast over bacteria. Antibiotic resistance genes are not transferred by yeasts unlike bacteria, and there has never been any evidence of their translocation [45].

*Pichia kudriavzevii* is present on the surfaces of fruits and vegetables and also in the soil [7]. *P. kudriavzevii* has the ability to generate toxin that can kill a variety of pathogenic microbes, consequently helping to preserve food [3]. Because of its capacity to digest cholesterol, this organism has been considered a possible probiotic [38]. This study aimed to evaluate the probiotic properties and the enzymes present in *P. kudriavzevii* isolated from home-made mango pickle.

## Methods

### Isolation and screening of yeast isolates

For the isolation of yeast isolates from traditional mango pickle, sample of pickle was homogenized with 0.9% NaCl solution and serial dilutions were plated on Yeast Malt agar (peptone—0.5%, yeast extract—0.3%, malt extract—0.3%, dextrose—1%, and agar—2.5%) procured from HiMedia, Mumbai, India, which was enriched with ampicillin (0.05 g/L) and acidified with 1N HCl to pH 5.0 to prevent bacterial growth. Plates were incubated at 30 °C for 48 h. Isolates were screened for different parameters (i.e., morphological, biochemical tests, carbohydrate fermentation (API 20C AUX), tolerance to acidic conditions and antimicrobial activity). Acid tolerance of the isolates was studied according to the method of Yu et al. [51]. Overnight grown yeast isolates were harvested (10,000 × g, 5 min, 4 °C), washed twice with PBS (phosphate buffer saline) buffer (pH 7.0). The cell pellet was then resuspended into PBS adjusted to pH 2, 3, and 7 (control) and incubated at 30 °C for 3 h. Survival of yeast isolates was calculated in terms of log cfu/mL. Antimicrobial effect of yeast isolates against three gram positive bacteria (*Bacillus cereus* MTCC 1272, *Staphylococcus aureus* subsp. *aureus* MTCC 96, *Listeria monocytogenes* MTCC 657) and five gram negative bacteria (*Pseudomonas aeruginosa* MTCC 424, *Escherichia coli* MTCC 118, *Shigella*, *Salmonella typhi* *Aeromonas hydrophilla*) were examined by agar well diffusion method [31]. Zone of inhibition was measured as millimeters (mm). Finally, yeast isolate Y33 was selected on the basis of acid tolerance and best antimicrobial activity for further study.

### Molecular identification of yeast isolate

The selected isolate was identified through isolation and amplification of the ITS DNA region at Biologia Research India Pvt. Ltd., New Delhi, India. Amplification of ITS region was done by using universal primers ITS1: TCCGTAGGTGAACCTGCGG and ITS4: TCCTCCGCTTATTGATATGC. The sequence was assembled, compared using BLAST, and submitted to GenBank database under accession number OM037458. A phylogenetic tree was constructed to determine the closest yeast species by the neighbor-joining approach, using MEGA 11.

### Evaluation of probiotic properties

Probiotic potential of *P. kudriavzevii* Y33 was examined for various factors viz., bile tolerance, cholesterol assimilation, cell surface hydrophobicity, autoaggregation, antibiotic susceptibility, exopolysaccharide production, and hemolytic activity.

### Bile tolerance

An overnight grown culture of selected yeast isolate was harvested (10,000 × g, 5 min, 4 °C) and washed twice with PBS buffer (pH 7.0). The cell pellet was resuspended into the bile solution containing 0.5%, 1%, and 2% ox bile (HiMedia) and incubated at 30 °C for 3 h [15]. The broth without bile solution was used as control. The results were expressed as log cfu/mL.

### Cholesterol assimilation

Cholesterol assimilation by yeast isolate was done using o-phthalaldehyde method according to the method given by Liong and Shah [24]. Three different bile salts viz. cholic acid, sodium taurocholate, and ox bile were used in the study. Cholesterol solution (Sigma-Aldrich) 10 mg/ml in 96% ethyl alcohol was made and sterilized using a filter. To simulate approximate levels in the digestive system, 70 μl of cholesterol solution was added to 10 ml of yeast peptone dextrose (YPD) broth (final cholesterol concentration of 70 μg/ml) with 0.2% (w/v) bile salts (ox bile/cholic acid/sodium taurocholate). YPD broth was inoculated with selected yeast isolate (1%) and incubated at 30 °C for 20 h. An uninoculated sample was used as control. After the incubation, the cells were centrifuged, and the leftover cholesterol concentration in the broth was quantified using Rudel and Morris [42] modified colorimetric method. Cholesterol assimilation was calculated as percentage by using formula:

$$A=100-\left(B/C\right)\times 100$$

where *A* = % of cholesterol removed, *B* = absorbance of the sample containing the cells, and *C* = absorbance of the sample without cells at 550 nm.

### Cell surface hydrophobicity

The ability of isolate to adhere to different hydrocarbons (cell surface hydrophobicity) was studied as per the method described by Rosenberg et al. [41]. The cell suspension was prepared as described in bile tolerance experiment and an optical density of 0.8 at 600 nm was adjusted. Then, equal volume of yeast suspension and hydrocarbons (n-hexadecane, xylene, and toluene) were mixed by vortexing for 5 min and allowed for phase separation by incubating at 37 °C for 1 h. The lower aqueous phase was withdrawn carefully, and the OD was determined. Hydrophobicity was calculated by using formula:

$$\mathrm{Hydrophobicity}\ \left(\%\right)=\left[\left(A0-A\right)/A0\right]\times 100$$

where *A*0 and *A* are absorbance before and after mixing with solvents at 600 nm.

### Autoaggregation

Autoaggregation ability of yeast isolate was performed by the modified method of Collado et al. [9]. Overnight grown cells were resuspended in PBS buffer and optical density of suspension was adjusted to 0.5 at 600 nm. The suspension (4 ml) was vortexed for 10 s and incubated at 30 °C. The autoaggregation ability was checked at different time intervals (0 h, 3 h, and 24 h), and autoaggregation ability was expressed in percentage by using formula as:

$$\mathrm{Autoaggregation}\ \left(\%\right)=\left[1- At/A0\right]\times 100$$

where *At* represents the absorbance at time *t* and A0 the absorbance at *t* = 0.

### Antibiotic susceptibility

For testing the antibiotic susceptibility of the yeast isolate, disk diffusion method was used [49]. Both antifungal and antibacterial antibiotics were used in the experiment. Antifungal agents used were ketoconazole (KT) (30 μg), clotrimazole (CC) (10 μg), itraconazole (IT) (30 μg), amphotericin-B (AP) (50 μg), nystatin (NS) (50 μg), and fluconazole (FLC) (10 μg), and antibacterial antibiotics used were vancomycin (VA) (30 μg), penicillin (P) (10 units), erythromycin (E) (15 μg), clindamycin (CD) (2 μg), and ampicillin (A) (10 μg).

### Exopolysaccharide production

For the evaluation of exopolysaccharide production, overnight grown yeast isolate was streaked on the ruthenium red milk agar plate (10% w/v, skim milk powder, 1% w/v, sucrose and 0.08 g/l ruthenium red, 1.5% w/v agar) and incubated at 30 °C for 24 h [34].

### Hemolytic activity

Yeast isolate (24 h grown culture) was streaked on the Columbia 5% Sheep Blood agar plate, and after 24 h of incubation at 30 °C, the plate was observed for the zone of clearance [25].

### Enzymatic profiling

The activity of different enzymes viz. β-galactosidase, protease, amylase, phytase, and lipase was checked in yeast isolate. For β-galactosidase activity, yeast culture was exponentially grown in YPD (yeast peptone dextrose) broth. Cells (50 μl) were treated with SDS (sodium dodecyl sulfate, 100 μl, 0.1M) for permeabilization; *o*-nitrophenyl β-galactopyranoside (100 μl; 0.1 M) (ONPG, HiMedia Mumbai, India) was used as substrate and incubated at 30 °C for 15 min. After the incubation, 1 ml of sodium carbonate (0.1 M) was added to stop the reaction and absorbance was recorded at 420 nm [30].

Supernatant of 48 h grown yeast isolate was used for protease assay. In assay, 150 μl of substrate (0.5% casein), supernatant (100 μl), and 750 μl of 50 mM Tris HCl buffer (pH 7) were added and incubated at 30 °C for 30 min. The reaction was stopped by adding 1 ml of 5% TCA (Trichloroacetic acid), reaction mixture was centrifuged, and absorbance was recorded at 275 nm [26].

To check the amylase activity of yeast isolate, 100 μl of substrate (0.5% starch), 100 μl of 48 h supernatant (enzyme), and 800 μl of acetate buffer (50 mM, pH 5.5) were incubated at 30 °C for 30 min. To stop reaction, 1 ml of DNS reagent was added to reaction mixture and then kept in boiling water for 5–10 min. After cooling, the absorbance was recorded at 540 nm [29].

Phytase activity of yeast isolate was assayed by determining the amount of phosphate liberated from sodium phytate. The reaction mixture comprising of 200 μl of enzyme and 800 μl of buffered substrate (30 mM sodium phytate in 0.25 M sodium acetate buffer pH 5.5) was incubated at 30 °C for 30 min. The reaction was quenched by adding 1 ml of 10% TCA, followed by 2 ml of coloring reagent (FeSO_4_.7H_2_O, 7.32%; ammonium molybdate, 1.5%; H_2_SO_4_, 4.8%). Optical density of the reaction mixture was determined at 750 nm [2].

The ability of yeast isolate to produce lipase enzyme was determined by photometric assay. In assay, 60 μl of *p*-nitrophenyl palmitate (30 mg p-NPP in 100ml of iso-propanol) as substrate, 40 μl of 24 h supernatant (enzyme), and 2.9 ml of Tris-HCl buffer (pH 8.0) were added and incubated at 37 °C for 30 min. The reaction was stopped by chilling at − 20 °C for 2 min. Absorbance was recorded at 410 nm [21]. Reaction buffers were used as blank during enzymatic reactions.

### Statistical analysis

The data was obtained from three separate experiments and provided as mean values. The statistical analysis was carried out with SPSS Inc. software (version 21.0). ANOVA and Tukey’s multiple comparison test (*p* < 0.05) were used to compare the results.

## Results

A total of 4 yeast isolates were isolated from home-made traditional mango pickle from Hamirpur district of Himachal Pradesh. The morphological properties (color, surface, margin and elevation), biochemical characteristics (catalase and urease test), and carbohydrate fermentation of yeast isolates are given in Table 1. The colonies of yeasts on YM agar plates were white/cream colored and one isolate Y33 with rough surface. All the four isolates were found to be negative for urease activity and only one isolate, i.e., Y44 showed catalase activity. Glucose was fermented by all four isolates, while some isolates also ferment glycerol, galactose, sorbitol, methyl-αD-glucopyranoside, N-acetyl-glucosamine, saccharose, maltose, and raffinose.

**Table 1:** Morphological, biochemical characteristics, and carbohydrate fermentation by yeast isolates

Characteristics	Yeast isolates			
	Y31	Y33	Y38	Y44
Color	White	White	Cream	White
Surface	Smooth	Rough	Smooth	Smooth
Margin	Entire	Elevated	Elevated	Elevated
Elevation	Convex	Convex	Convex	Convex
Catalase	**−**	**−**	**−**	+
Urease test	**−**	**−**	**−**	**−**
Carbohydrate fermentation				
D-glucose	**+**	**+**	**+**	**+**
Glycerol	**−**	**+**	**+**	**−**
Calcium 2-keto-gluconate	**−**	**−**	**−**	**−**
L-arabinose	**−**	**−**	**−**	**−**
D-xylose	**−**	**−**	**−**	**−**
Adonitol	**−**	**−**	**−**	**−**
Xylitol	**−**	**−**	**−**	**−**
D-galactose	**+**	**−**	**+**	**+**
Inositol	**−**	**−**	**−**	**−**
D-sorbitol	**+**	**−**	**−**	**−**
Methyl-αD-glucopyranoside	**+**	**−**	**+**	**+**
N-acetyl-glucosamine	**−**	**+**	**−**	**−**
D-cellobiose	**−**	**−**	**−**	**−**
D-lactose	**−**	**−**	**−**	**−**
D-maltose	**−**	**−**	**−**	**+**
D-saccharose	**+**	**−**	**+**	**+**
D-trehalose	**−**	**−**	**−**	**−**
D-melezitose	**−**	**−**	**−**	**−**
D-raffinose	**+**	**−**	**−**	**+**

+ presence, − absence of enzyme/fermentation

**Fig. 1:**
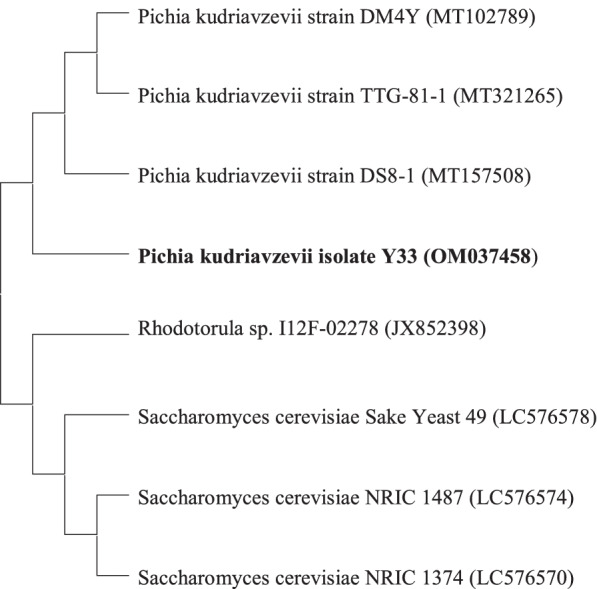
Phylogenetic tree of *P. kudriavzevii* Y33 based on homology of ITS gene sequences. Toluene showed significantly high cell surface hydrophobicity at *p* < 0.05 as measured by 2 sided Tukey’s post hoc range test between replications

The acid tolerance and antimicrobial activity of all the four isolates are shown in Table 2 and Table 3. Among all the isolates, Y33 showed high acid tolerance as well as broad and strong antimicrobial activity against all the tested pathogens ranging from 10 to 22 mm zone of inhibition. Therefore, on the basis of high tolerance and strongest antimicrobial activity, Y33 was selected for further studies. After sequencing, sequences of *P. kudriavzevii* Y33 with accession number OM037458 were submitted to GenBank database. The phylogenetic analysis of *P. kudriavzevii* Y33 was also performed by neighbor-joining approach by using MEGA 11 software (Fig. 1).

**Table 2:** Acid tolerance (log cfu/ mL) of yeast isolates after 3 h of incubation

pH	Viability (log cfu/mL)^d^				% survival of isolate^e^			
	Y31	Y33	Y38	Y44	Y31	Y33	Y38	Y44
**pH 7 (control)**	6.40^c^ ± 0.45	6.50^b^ ± 0.06	6.70^c^ ± 0.23	6.90^c^ ± 0.02	100	100	100	100
**pH 3**	5.70^b^ ± 0.20	6.18^ab^ ± 0.57	5.48^b^ ± 0.19	5.87^b^ ± 0.14	89.10	95.08	81.79	85.07
**pH 2**	5.00^a^ ± 0.04	5.76^a^ ± 0.04	4.67^a^ ± 0.54	5.10^a^ ± 0.20	78.13	88.62	69.70	73.91

^a−c^Average in the columns with same superscript letter are not significantly (*p* < 0.05) different as measured by 2 sided Tukey’s post hoc range test between replications

^d^Values represented as mean ± standard deviation (SD) of triplicate analysis

^e^% survival of isolate = (log cfu at 3 h/log cfu at 0 h) × 100

**Table 3:** Antimicrobial activity (mm) of yeast isolates against various pathogenic organisms

	Y31	Y33	Y38	Y44
***S. typhi*(mm)**	-	10.50 ± 1.50	-	-
***E. coli*(mm)**	-	10.00 ± 2.00	-	8.00 ± 1.00
***Shigella*(mm)**	-	13.50 ± 1.50	9.00 ± 1.50	-
***P. aeruginosa*(mm)**	-	14.00 ± 1.00	-	7.50 ± 2.50
***B. cereus*(mm)**	8.00 ± 2.50	12.50 ± 0.50	-	-
***S. aureus*(mm)**	-	13.50 ± 0.50	11.00 ± 0.50	-
***A. hydrophilla*(mm)**	12.00 ± 1.50	22.00 ± 2.00	10.00 ± 1.50	14.00 ± 0.50
***L. monoctyogenes*(mm)**	-	10.50 ± 1.50	-	-

Values represented as mean ± standard deviation (SD) of triplicate analysis, - no zone of inhibition

### Probiotic properties

Probiotic traits of *P. kudriavzevii* Y33 were studied. These include bile tolerance, cholesterol assimilation, cell surface hydrophobicity, autoaggregation, antibiotic susceptibility, exopolysaccharide production, and hemolytic activity.

### Bile tolerance

When exposed to the bile salt, *P. kudriavzevii* Y33 showed no significant difference (*p* < 0.05) at different concentration of bile. No significant loss of viability was observed even at 2% of bile concentration as compared to control. *P. kudriavzevii* Y33 showed high viability index, i.e., 97 and 95% at concentration of 1 and 2% of bile, respectively (Table 4).

**Table 4:** Bile tolerance of *P. kudriavzevii* Y33 after 3 h of incubation

Bile concentration	Viability (log cfu/mL)^b^	% survival of isolate^c^
Control	6.64^a^ ± 0.34	100
0.5%	6.45^a^ ± 0.09	97.14
1%	6.44^a^ ± 0.17	96.99
2%	6.35^a^ ± 0.25	95.63

^a^Average in the columns with same superscript letter are not significantly (*p* < 0.05) different as measured by 2 sided Tukey’s post hoc range test between replications

^b^Values represented as mean ± standard deviation (SD) of triplicate analysis

^c^% survival of isolate = (log cfu at 3 h/log cfu at 0 h) × 100

### Cholesterol assimilation

During the 20 h growth of *P. kudriavzevii* Y33, the assimilation of cholesterol was tested using three bile salts, cholic acid (deconjugated bile), taurocholate (conjugated bile), and ox bile (containing both conjugated and deconjugated bile). Overall cholesterol assimilation was best in the medium containing ox bile and taurocholate, at 88.58 ± 0.31 and 86.83 ± 0.83% of its initial content, respectively, whereas least cholesterol assimilation was found with cholic acid (5.15 ± 0.26%) for *P. kudriavzevii* Y33 (Table 5).

**Table 5:** Cholesterol assimilation (%) of *P. kudriavzevii* Y33 in different bile salts

Isolate	Ox bile	Cholic acid	Taurocholate
*P. kudriavzevii* Y33	88.58^c^ ± 0.31	5.15^a^ ± 0.26	86.83^b^ ± 0.83

Values represented as mean ± standard deviation (SD) of triplicate analysis

^a-c^Average in the rows with same superscript letter are not significantly (p < 0.05) different as measured by 2 sided Tukey’s post hoc range test between replications

### Cell surface hydrophobicity

**Fig. 2:**
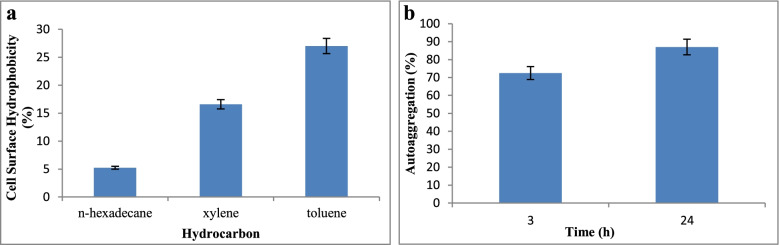
**a** Cell surface hydrophobicity. **b** Autoaggregation of *P. kudriavzevii* Y33

The cell surface hydrophobicity of *P. kudriavzevii* Y33 was evaluated by microbial adhesion to different hydrocarbons (n-hexadecane, xylene, and toluene). The percentage of cell surface hydrophobicity is given in Fig. 2a. *P. kudriavzevii* Y33 showed 27.02% cell surface hydrophobicity towards toluene and 16.60% towards xylene.

### Autoaggregation

The results showed that the autoaggregation ability of yeast isolate increases with increasing the time of incubation. *P. kudriavzevii* Y33 showed strongest autoaggregation ability, more than 87% after 24 h and 72.45% after 3 h of incubation (Fig. 2b).

### Antibiotic susceptibility

One of the most desirable qualities of a potential probiotic organism is that it must be safe for human consumption, and no reports of eukaryotic probiotics transferring antibiotic resistance genes have been found. The yeast isolate has been found sensitive to different antifungal agents and resistant to antibacterial antibiotics except erythromycin (Table 6).

**Table 6:** Antibiotic susceptibility profile of *P. kudriavzevii* Y33

Isolate	KT (mm)	CC (mm)	IT (mm)	AP (mm)	NS (mm)	FLC (mm)	VA (mm)	P (mm)	E (mm)	CD (mm)	A (mm)
*P. kudriavzevii* Y33	22	24	26	10	20	17	R	R	12	R	R

Ketoconazole (KT), clotrimazole (CC), itraconazole (IT), amphotericin-B (AP), nystatin (NS), fluconazole (FLC), vancomycin (VA), penicillin (P), erythromycin (E), clindamycin (CD), ampicillin (A)

*R* resistant

### Exopolysaccharide production and hemolytic activity

*P. kudriavzevii* Y33 produced exopolysaccharide on ruthenium red plates after 24 h of incubation. No hemolysis was observed when the isolate was streaked on Columbia 5% sheep blood agar plates which validate the safety of the yeast isolate.

### Enzymatic profiling

Probiotics produce a number of useful enzymes that aid in food digestion and hence boost human health. Therefore, *P. kudriavzevii* Y33 was evaluated for various enzymes such as β-galactosidase, amylase, protease, phytase, and lipase, and the results are given below:

β-galactosidase activity is an important characteristic in probiotic strains. Its deficiency is associated to the inability to breakdown lactose in the intestine [35]. In the present study, *P. kudriavzevii* Y33 was found to possess β-galactosidase activity which is 0.054 U/mg.

**Fig. 3:**
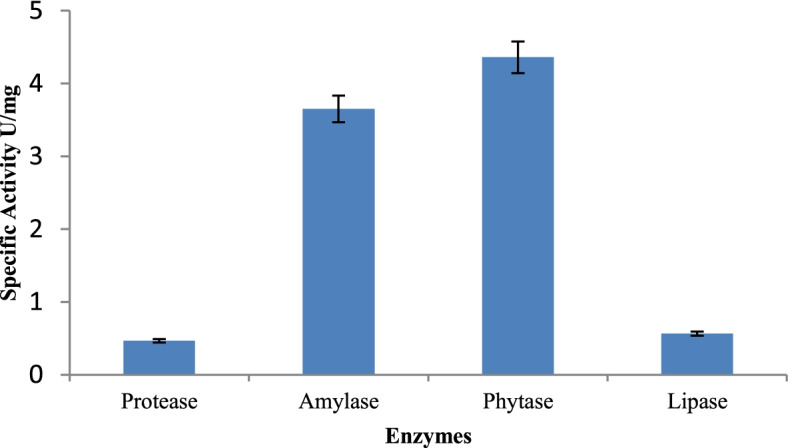
Proteolytic, amylase, phytase, and lipase activity of *P. kudriavzevii* Y33

In quantitative screening of proteolytic activity, the selected isolate showed specific activity of 0.46 U/mg (Fig. 3). The proteolytic activity of probiotics is important as the hydrolysis of proteins is required to meet the amino acids/nitrogen requirement of the microorganisms during fermentation process.

*P. kudriavzevii* Y33 was found to possess high amylase activity with specific activity of 3.64 U/mg. Amylase hydrolyzes starch and the resulting sugars are used by microorganisms as a carbon and energy source during fermentation.

The main storage form of phosphorus is phytate in plants; the phytase enzyme liberates the free inorganic phosphorous and improves the nutritional value of food and feed. The addition of phytase to food is also considered as a method to enhance the bioavailability of essential dietary minerals. Specific activity of *P. kudriavzevii* Y33 for phytase was observed to be 4.36 U/mg (Fig. 3).

Although many microorganisms are capable of producing lipases, lipase production is prevalent among yeasts. Triglycerides are a major source of emulsified substrates for lipases. In *P. kudriavzevii* Y33 specific activity for lipase was observed to be 0.564 U/mg.

## Discussion

Although bacteria are the most common microbes in traditional fermented foods, yeasts coexist with bacteria in many naturally fermented foods and beverages [13]. Some yeasts interact with bacteria to help them survive and flourish, and their interactions may have an impact on fermentation [22]. In fermentation, yeast plays key functions such as alcohol production, preservation by reducing the pH and production of killer toxin, texture improvement by leavening, improved nutritive values and removal of anti-nutritive factors, value addition by developing bioactive peptides, and vitamin production.

In the present study, the yeasts were isolated from a home-made mango pickle. Yeast isolate Y33 was chosen for the present study after morphological, biochemical, sugar fermentation, acid tolerance, and antimicrobial activity tests. The selected yeast isolate was identified using the ITS gene method, and after sequencing, the sequences of *Pichia kudriavzevii* Y33 were submitted to the GenBank database with an accession number OM037458. *P. kudriavzevii* was mostly identified from different fermented foods like cheese, *kimchi* [16, 33], and fermented cocoa beans [11]. *P. kudriavzevii* isolated from the food environment could be a potential probiotic [40].

Microorganisms considered for use as probiotics must first overcome the hostile environment of the human gastrointestinal tract (GIT) before colonization. Probiotics should be acid, lysozyme, and bile resistant in order to reach active and viable levels through the GIT. As a result, for the selection of highly efficient probiotic strains, in vitro assessment of safety and functional qualities is important. Probiotics must be able to withstand acidic pH and remain viable in the presence of stomach fluids. The results of present study revealed that *P. kudriavzevii* Y33 at pH 3 showed equal percent of viability as at pH 7. However, incubation at pH 2 results in significant decrease in the viability of isolate. Yeasts have varying ability to survive and develop in low pH environments. Yeast strains isolated from cocoa fermentation could survive at pH 3 [50]. The strains of *P. kudriavzevii* isolated from “*ogi*,” a typical Nigerian fermented dish were found to survive even at pH 2, the common pH of the gastric juice [1].

The ability of probiotics to suppress the growth of pathogenic organisms is one of its most important properties. The yeast can inhibit pathogens from binding to enterocytes, by exerting a direct antagonistic impact and/or secreting a variety of metabolites and enzymes. Furthermore, pathogenic microbes compete with *S. cerevisiae* var. *boulardii* for food and mucosal receptors in GI tract, preventing pathogens from colonizing the gut of the host [12]. Instead of attaching to enterocytes, pathogens rapidly bind to the mannans on the surface of yeast [14] and are then removed from the host's digestive system after being agglutinated by yeast. In the present study, *P. kudriavzevii* Y33 showed strong antimicrobial activity against all the tested pathogens. The inhibitory effect of isolate is either by lowering pH, producing specific organic acids, or competing for substrates and space. Similar study was reported by Bajwa and Sharma [4], where yeasts isolated from *Sidra* a fermented fish also showed the broadest antimicrobial activity against pathogenic bacteria.

Bile salts are antimicrobial agents that can harm microorganisms by destroying bacterial membranes, denaturing proteins, chelating iron and calcium, and causing DNA damage. The optimal bile salts concentration in the human gastrointestinal tract is between 0.3 and 0.6%. The studied isolate showed significant viability (95%) even at 2% of bile concentration when compared to the control. According to Helmy et al. [16], probiotic yeast *P. kudriavzevii* QLB from *Karish* cheese demonstrated tolerance to the bile concentration of 2%.

Increase in the blood cholesterol levels are thought to be the most important risk factor for chronic illnesses like coronary heart disease. Studies suggested that probiotics have potential to lower the level of blood cholesterol. These microorganisms decrease the cholesterol through a variety of processes, including cholesterol assimilation by microorganisms, bile acid deconjugation, and cholesterol adhesion to the cell wall of bacteria [44]. The present study revealed that *P. kudriavzevii* Y33 showed highest cholesterol assimilation in ox bile (88.58%) and taurocholate (86.83%). Kathade et al. [20] revealed that *P. kudriavzevii* from the gut of the edible freshwater snail “*Pila globosa*” showed ability to remove cholesterol from media.

Probiotics must have a high cell surface hydrophobicity and autoaggregation capability in order to colonize and adhere to the intestinal epithelium. The high adherence property of probiotics assist their gastric motility and improves the interactions between probiotic and the host [23]. In this context, *P. kudriavzevii* Y33 was screened for cell surface hydrophobicity and autoaggregation, and the isolate showed 27% degree of hydrophobicity towards toluene and strong autoaggregation, i.e., 87% after 24 h of incubation. It has been found that microbial adhesion to cell surface is more strongly related to autoaggregation as compared to the hydrophobicity [10]; as a result, a high level of autoaggregation ability may be responsible for adherence property of this isolate. A similar result was observed by Kathade et al. [20], where *P. kudriavzevii* showed 93% autoaggregation property. In an another study, after 2 h of incubation, autoaggregation percentages of *P. kudriavzevii* isolated from traditional soft cheese ranged from 80.76% to 87.32% [28]. Self-recognizing surface features like surface-bound proteins and exopolysaccharides play a part in autoaggregation ability [48].

The second major safety consideration when using bacteria as probiotics is determining their antibiotic susceptibility. Antibiotic resistance as well as the absence of transferable antibiotic resistance genes in probiotics is critical in reducing the risk of antibiotic resistance genes spreading and ensuring the safety of probiotics use [37]. In the present study, *P. kudriavzevii* Y33 was found to be resistant to most of the antibiotics, while it was sensitive to the antifungal agents. Antibiotic resistance reported in *S. boulardii* and *Lactobacillus* strains were supposed to be intrinsic or spontaneous and thus non-transmissible [17]. *P. kudriavzevii* isolated from the edible freshwater snail showed resistance to most of the antibiotics used in study [20].

Exopolysaccharides are polymeric molecules that aid in the colonization of probiotics in the intestinal mucosa [19]. Exopolysaccharide are the key elements of bacterial biofilms and impact on bacterial adhesion to host cells has been widely characterized [8]. *P. kudriavzevii* Y33 was found to produce the exopolysaccharide during study.

The ability of yeast strains to lyse red blood cells by making a clear zone on blood agar can be used to assess their pathogenicity. Hemolysis is categorized into three types: α-hemolysis, β-hemolysis, and γ-hemolysis. Alpha-hemolysin causes partial damage to red blood cells, beta-hemolysin causes complete lysis of red blood cells, and gamma-hemolysis causes no harm to red blood cells [32]. No hemolysis was observed in the present study. A total of 22 yeast isolates including *P. kudriavzevii* isolated from cocoa fermentation were found to be gamma hemolytic and categorized as non-pathogenic and suitable for use as probiotics [50]. Menezes et al. [27] revealed that yeasts isolated from fermented foods are basically non-hemolytic.

Probiotics benefit the host’s health in a number of ways, one of which is by releasing enzyme that increase nutrition utilization in the intestine [46]. The extracellular enzymatic profiles of yeasts isolated from the tropical habitats of the Brazilian rain forest were investigated, and it was found that they were a source of industrially relevant enzymes such as amylases, esterases, lipases, proteases, pectinases, and chitinases [5]. In the present study, *P. kudriavzevii* Y33 was observed to produce several enzymes such as β-galactosidase, protease, amylase, phytase, and lipase. During a study, Syal and Vohra [46] revealed that seven yeasts isolated from *idli* and *jalebi* batter were found to produce β-galactosidase, phytase, protease, and lipase, and none of the strains showed amylase production.

## Conclusions

The results of present study of *P. kudriavzevii* Y33 isolated from traditionally home-made mango pickle revealed that the strain could be good probiotic candidate since it possesses promising qualities that are needed in probiotics. Further, in vivo investigations in humans and animals are needed to evaluate the functionality of the strain. The strain could be given to patients who are on long-term antibiotic therapy to protect them from food-borne enteric infections. *P. kudriavzevii* Y33 have the ability to breakdown anti-nutrients like phytic acid and tannic acid, hence increasing dietary nutrition. The beneficial properties of *P. kudriavzevii* Y33 indicate that it could be a potential probiotic candidate and could be widely employed as a food and feed additive.

## Data Availability

Not applicable.
